# Supplementary material for: Boron encapsulated in a liposome can be used for combinational neutron capture therapy
Source: Nat Commun. 2022 Apr 19;13:2143. doi: 10.1038/s41467-022-29780-w (PMC9018847; doi:10.1038/s41467-022-29780-w)
Supplement: Supplementary file 1 — Supplementary Information [file 41467_2022_29780_MOESM1_ESM.pdf]

Supporting online material for

Boron encapsulated in a liposome can be used for Combinational Neutron Capture Therapy

Jiyuan Li[#1], Qi Sun[#2], Chuanjie Lu[1], Han Xiao[3], Zhibin Guo[1], Dongban Duan[1], Zizhu Zhang[4,5], Tong Liu[5] and Zhibo Liu*[1,2]

[1]Beijing National Laboratory for Molecular Sciences, Radiochemistry and Radiation Chemistry Key Laboratory of Fundamental Science, College of Chemistry and Molecular Engineering, Peking University, Beijing 100871 (China)

[2]Peking University-Tsinghua University Center for Life Sciences, Beijing, 100871 (China)

[3]University of Chinese Academy of Sciences Beijing, 100049 (China)

[4]Beijing Nuclear Industry Hospital, Beijing, 100045 (China)

[5]Beijing Capture Tech Co., Ltd, Beijing, 102413 (China)

[#]The authors contributed equally to this paper.

[*]To whom correspondence should be addressed. E-mail Z.L (zbliu@pku.edu.cn)

The file includes:

1) Supplementary Table 1 to 6

2) Supplementary Figure 1 to 11

3) NMR information (Description and Supplementary Figure 12-23)

4) HRMS information (Description and Supplementary Figure 24-27)

Supplementary tables and figures

Supplementary Table 1 No-Bonds parameter of MD simulations.

Type	C6	C12
H	0.0000E+00	0.0000E+00
HC	8.4640E-05	1.5129E-08
B	2.3406E-03	4.9373E-06
C	2.3406E-03	4.9373E-06
S	9.9840E-03	1.3075E-05

Supplementary Table 2 Bonds parameter of MD simulations.

Bonds	b_0(Å)	kb
H-$\boldsymbol{B_\alpha}$	1.19	2.6481e+07
H-$\boldsymbol{B_\beta}$	1.19	2.6481e+07
H-$\boldsymbol{B_\gamma}$	1.18	3.5909e+07
H-$\boldsymbol{B_\delta}$	1.18	3.5909e+07
H-C	1.09	1.2300e+07
S-C	1.67	3.0478e+06
C-C	1.63	8.5653e+05
C-$\boldsymbol{B_\alpha}$	1.63	8.7100e+06
C-$\boldsymbol{B_\beta}$	1.63	4.7200e+06
$\boldsymbol{B_\alpha}$-$\boldsymbol{B_\beta}$	1.78	1.0665e+06
$\boldsymbol{B_\alpha}$-$\boldsymbol{B_\gamma}$	1.76	1.8519e+06
$\boldsymbol{B_\beta}$-$\boldsymbol{B_\beta}$	1.79	5.6200e+06
$\boldsymbol{B_\beta}$-$\boldsymbol{B_\gamma}$	1.78	2.6424e+06
$\boldsymbol{B_\beta}$-$\boldsymbol{B_\delta}$	1.78	2.7200e+06
$\boldsymbol{B_\gamma}$-$\boldsymbol{B_\delta}$	1.79	2.1847e+06
$\boldsymbol{B_\delta}$-$\boldsymbol{B_\delta}$	1.69	1.9257e+06

Supplementary Table 3 Angles parameter of MD simulations.

Angles	θ	Ka
S-C-$\boldsymbol{B_{\alpha}}$	114.00	1559.41
S-C-$\boldsymbol{B_{\beta}}$	132.00	760.00
S-C-C	120.00	560.00
C-$\boldsymbol{B_{\alpha}}$-C	56.39	2100.95
C-$\boldsymbol{B_{\alpha}}$-$\boldsymbol{B_{\beta}}$	124.00	730.00
HC-C-$\boldsymbol{B_{\alpha}}$	118.00	7474.41
HC-C-$\boldsymbol{B_{\beta}}$	125.00	375.00
HC-C-C	120.00	505.00
H-$\boldsymbol{B_{\alpha}}$-C	120.00	390.00
H-$\boldsymbol{B_{\alpha}}$-$\boldsymbol{B_{\beta}}$	132.22	390.00
H-$\boldsymbol{B_{\beta}}$-$\boldsymbol{B_{\alpha}}$	119.00	2211.40
H-$\boldsymbol{B_{\beta}}$-$\boldsymbol{B_{\beta}}$	119.74	3398.91
H-$\boldsymbol{B_{\beta}}$-$\boldsymbol{B_{\gamma}}$	121.00	685.00
H-$\boldsymbol{B_{\beta}}$-$\boldsymbol{B_{\delta}}$	132.00	760.00
H-$\boldsymbol{B_{\gamma}}$-$\boldsymbol{B_{\delta}}$	121.00	685.00
H-$\boldsymbol{B_{\gamma}}$-$\boldsymbol{B_{\beta}}$	117.00	635.00
H-$\boldsymbol{B_{\delta}}$-$\boldsymbol{B_{\gamma}}$	121.00	375.00
H-$\boldsymbol{B_{\delta}}$-$\boldsymbol{B_{\delta}}$	121.40	690.00
$\boldsymbol{B_{\alpha}}$-C-$\boldsymbol{B_{\alpha}}$	115.71	1201.92
$\boldsymbol{B_{\alpha}}$-$\boldsymbol{B_{\beta}}$-$\boldsymbol{B_{\beta}}$	97.40	469.00
$\boldsymbol{B_{\alpha}}$-$\boldsymbol{B_{\beta}}$-$\boldsymbol{B_{\gamma}}$	60.30	531.36
$\boldsymbol{B_{\alpha}}$-$\boldsymbol{B_{\beta}}$-$\boldsymbol{B_{\delta}}$	103.00	420.00
$\boldsymbol{B_{\beta}}$-$\boldsymbol{B_{\alpha}}$-$\boldsymbol{B_{\beta}}$	106.75	503.00
$\boldsymbol{B_{\beta}}$-$\boldsymbol{B_{\beta}}$-$\boldsymbol{B_{\gamma}}$	107.57	484.00
$\boldsymbol{B_{\beta}}$-$\boldsymbol{B_{\beta}}$-$\boldsymbol{B_{\delta}}$	59.90	508.93
$\boldsymbol{B_{\beta}}$-$\boldsymbol{B_{\gamma}}$-$\boldsymbol{B_{\delta}}$	60.30	531.36
$\boldsymbol{B_{\beta}}$-$\boldsymbol{B_{\delta}}$-$\boldsymbol{B_{\gamma}}$	59.90	508.93
$\boldsymbol{B_{\gamma}}$-$\boldsymbol{B_{\delta}}$-$\boldsymbol{B_{\delta}}$	63.30	2254.27

Supplementary Table 4 Bilayer width and maximum density of BoPs by MD simulations.

BoPs	Bilayer width (nm)	Maximum density (kg/m^3)
DPPC	4.83	1484.07
BoP-1	3.93	1509.98
BoP-2	4.65	1446.07
BoP-3	5.24	1382.70
BoP-4	5.60	1466.98

Supplementary Table 5 Biodistribution data and tumor-to-normal tissue ratio (T/N ratio) of boronsome in 4T1 bearing mice measured by ICP-OES 12 h post once injection of boronsome (500 mg/kg, i.v.). Data are means ± SEM (n = 4 mice).

Tissues	Boron concentration (ppm)	T/N ratio
Tumor	93.25±2.7	-
Lung	1.50±0.1	63.59±2.3
Muscle	2.60±0.1	37.30±1.8
Brain	1.25±0.1	82.71±6.4
Fat	3.65±0.1	25.85±0.7
Bone	5.90±0.3	16.32±08
Heart	9.85±0.3	9.58±0.3
Blood	19.15±0.5	4.92±0.1
Liver	351.71±10.0	0.27±0.0
Spleen	205.45±8.0	0.47±0.0

Supplementary Table 6 Dose computations by Monte Carlo simulation using Simulation Environment for Radiotherapy Applications (SERA) system. The beam location is determined by the source geometry, the target point (X, Y, Z) represents the distance from the target to the source center. For dose estimation in BNCT, all of these dose components including B-10, gamma, N-14 and hydrogen need to be calculated.

X (cm)	Y (cm)	Z (cm)	reg	Total dose rate (cGy/s)	B-10 dose rate (cGy/s)	Gamma dose rate (cGy/s)	N-14 dose rate (cGy/s)	Hydrogen dose rate (cGy/s)	Total dose (Gy)
0	-4.5	-0.00100	air	0.0372	0.0000	0.0167	0.0123	0.0082	0.6692
0	-4.5	-0.00135	tumor tissue	0.1014	0.0642	0.0167	0.0123	0.0082	1.8254
0	-4.5	-0.25140	tumor tissue	0.1141	0.0718	0.0189	0.0138	0.0097	2.0542
0	-4.5	-0.50120	normal tissue	0.0569	0.0128	0.0235	0.0099	0.0106	1.0236
0	-4.5	-0.75120	normal tissue	0.0547	0.0115	0.0236	0.0090	0.0106	0.9841
0	-4.5	-1.00100	normal tissue	0.0376	0.0061	0.0173	0.0048	0.0094	0.6769
0	-4.5	-1.25100	normal tissue	0.0337	0.0051	0.0153	0.0041	0.0093	0.6071
0	-4.5	-1.50100	normal tissue	0.0237	0.0030	0.0081	0.0024	0.0102	0.4271
0	-4.5	-1.70000	air	0.0194	0.0000	0.0068	0.0022	0.0103	0.3488
0	-4.5	-1.95000	air	0.0181	0.0000	0.0054	0.0021	0.0105	0.3256
0	-4.5	-2.00100	air	0.0175	0.0000	0.0038	0.0029	0.0107	0.3146

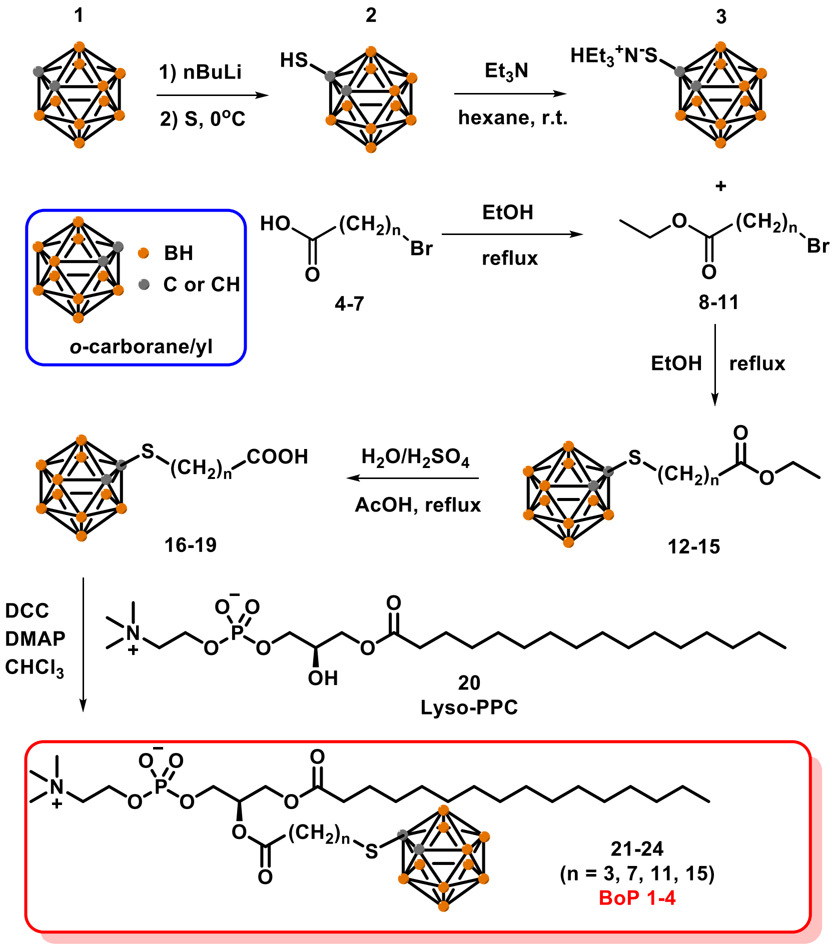

Supplementary Figure 1. Chemical synthesis of BoPs. Reagents and solvents were purchased from Sigma-Aldrich, Thermo Fisher or Novabiochem. All nuclear MR spectra were recorded at room temperature on a Bruker Avance 400 MHz or 500 MHz spectrometer. Signals are presented as ppm, and multiplicity identified as s = single, br = broad, d = doublet, t = triplet, q = quartet, m = multiplet; coupling constants in Hz. Concentration under reduced pressure was performed by rotary evaporation. Chemistry yields refer to isolated pure chemicals.

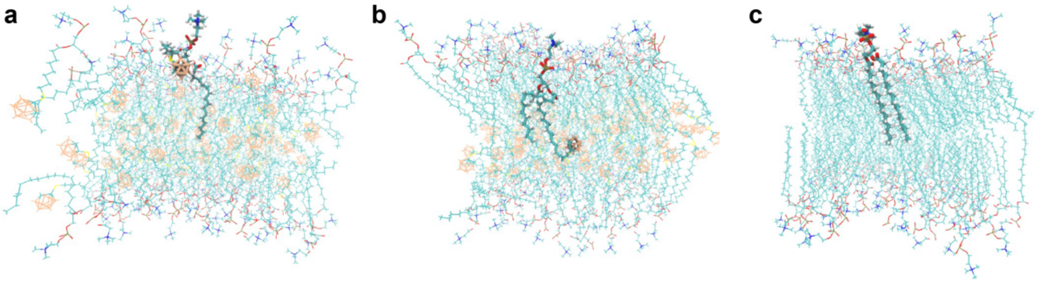

Supplementary Figure 2. Cross-section of liposomes by MD simulations. **a** BoP-2. **b** BoP-4. **c** DPPC.

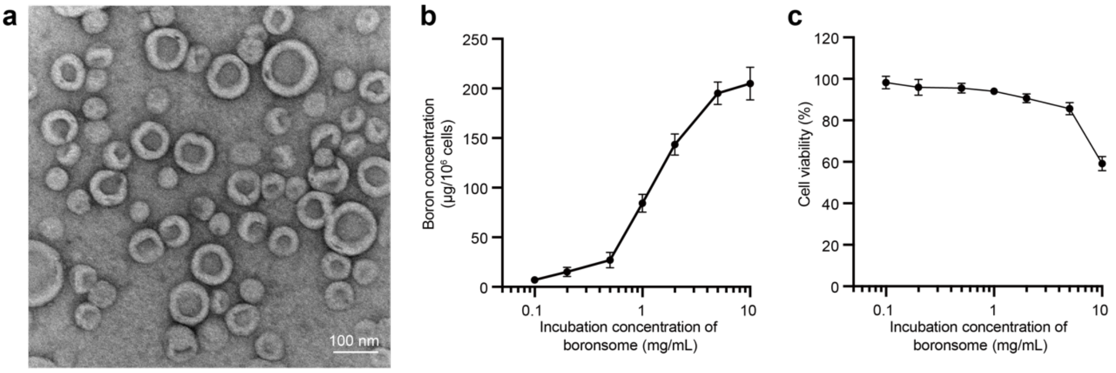

Supplementary Figure 3. Boronsome presents uptake in line with clinical needs and exhibited good tolerance. **a** Imaging of boronsomes by transmission electron microscopy (TEM). Three independent experiments were performed and representative results are shown. Scale bar, 100 nm. **b** Cellular boron uptake of 4T1 cells incubated with various concentrations of boronsome for 24 h (n = 4). **c** Cell viability of 4T1 cells incubated with various concentrations of boronsome for 24 h (n = 6). Data are means ± SEM. Source data are provided as a Source Data file.

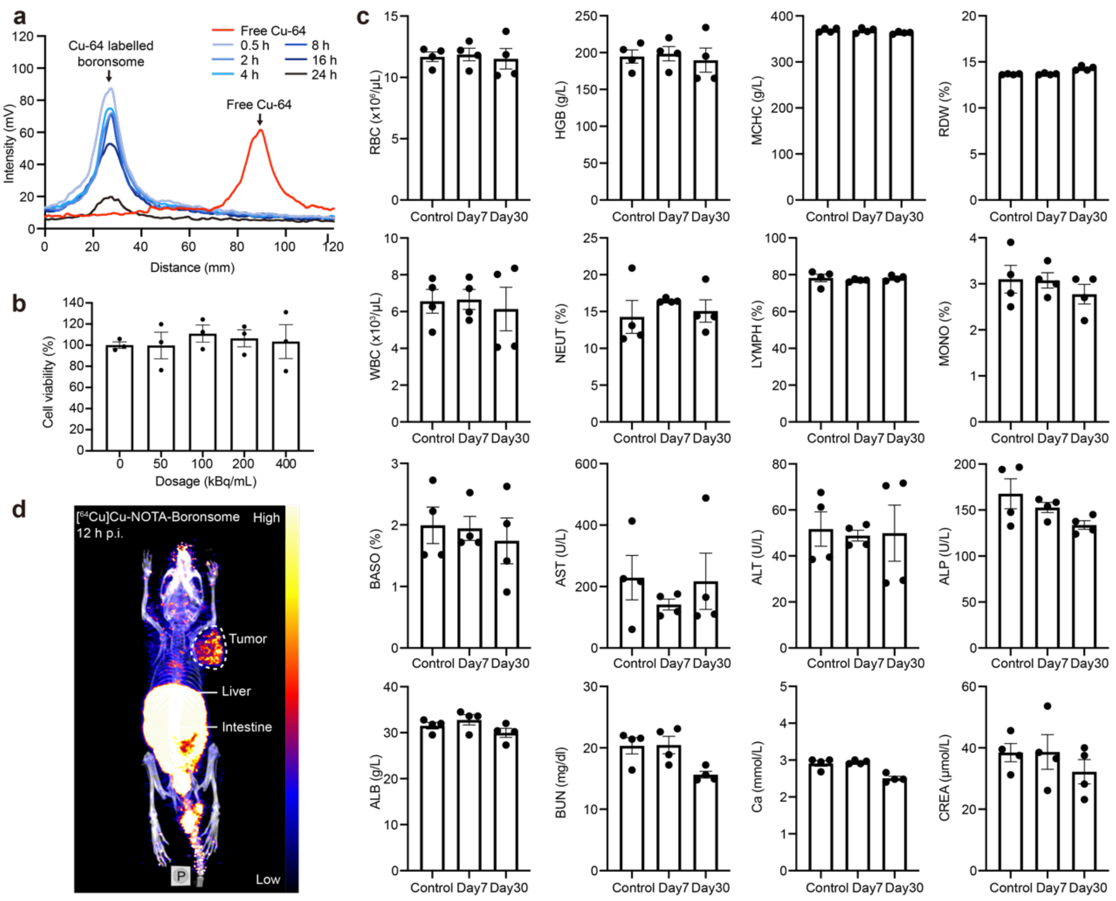

Supplementary Figure 4. Stability and biocompatibility of [^{64}Cu]Cu-NOTA-boronsomes. a Radio thin layer chromatography (radio-TLC) for the conformation of notable stability of [^{64}Cu]Cu-NOTA-boronsomes. **b** Survival fraction (n = 3) of 4T1 cells under various dosages of [^{64}Cu]Cu-NOTA-boronsome after 24 h assessed with a CCK-8 assay. We picked a dosage (40 kBq, 2 x 10^4 cells) that far exceeds 1000-fold the activity of tumour region (200 kBq, about 10^8 cells), and exceed (300-fold) the activity of liver region (2000 kBq, about 3 x 10^8 cells), which has a highest uptake of [^{64}Cu]Cu-NOTA-boronsome according to the PET study. **c** The blood routine and biochemical test of 4T1 tumor-bearing mice intravenously injected with [^{64}Cu]Cu-NOTA-boronsome at day 7 and day 30 (n = 4). **d** Whole-body maximum intensity projection PET images of a 4T1 bearing mouse, 12 h after intravenous injection with [^{64}Cu]Cu-NOTA-boronsome. Tumors were circled with white dashed line. Data are means ± SEM. Source data are provided as a Source Data file.

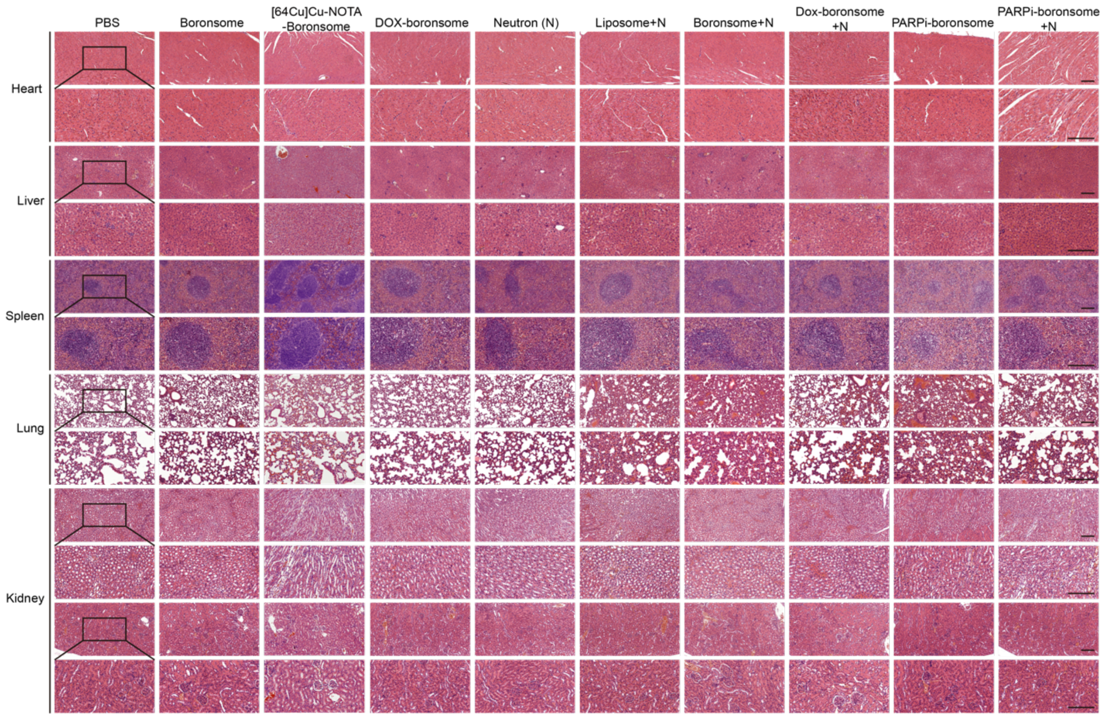

Supplementary Figure 5. Hematoxylin and eosin (H&E) staining of hearts, livers, spleens, lungs and kidneys obtained from 4T1 tumor bearing mice. Three independent experiments were performed and representative results are shown. Scale bar, 200 μm.

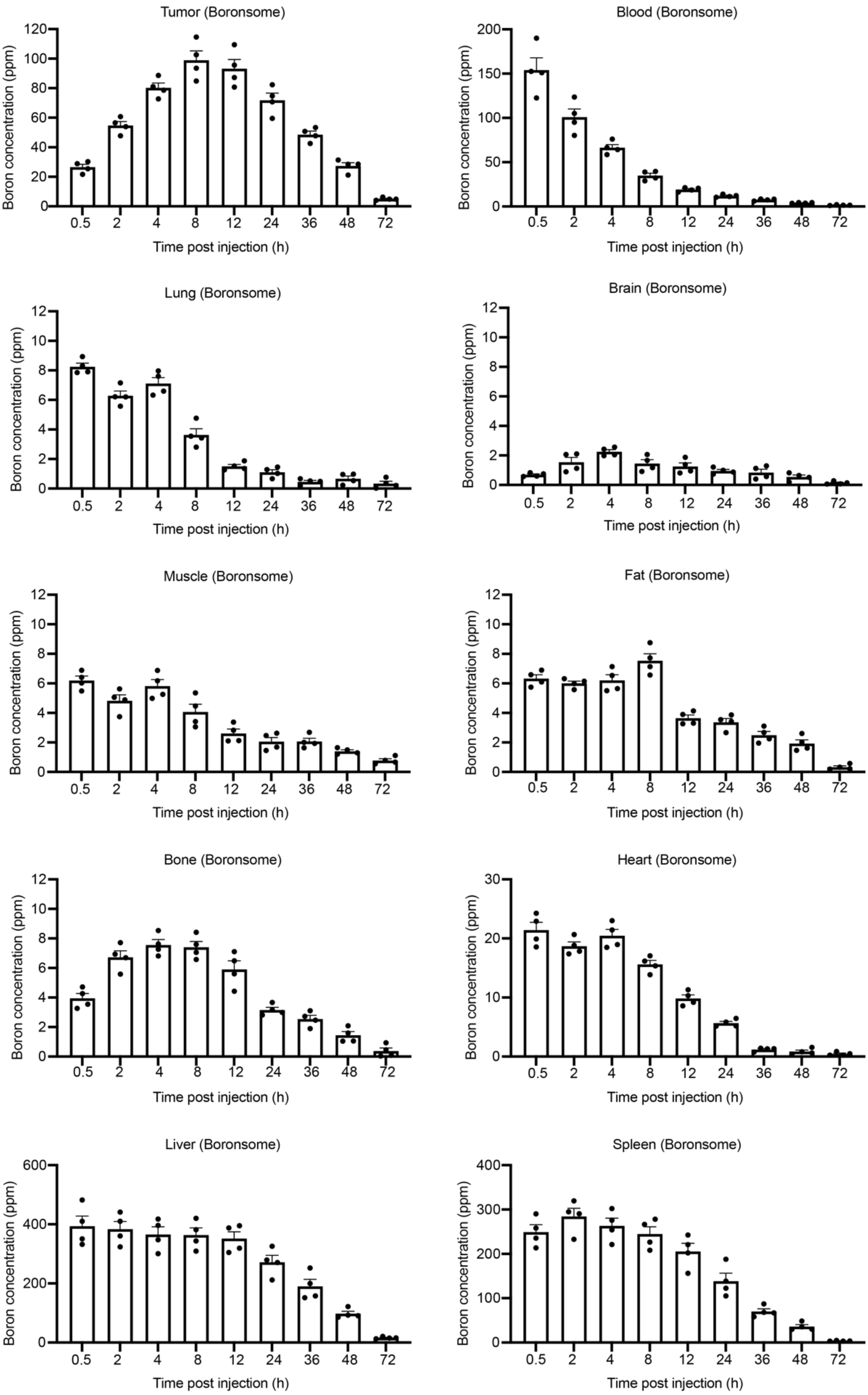

Supplementary Figure 6. Time-dependent boron concentration in tumor, blood, lung, brain, muscle, fat, bone, heart, liver, and spleen post once injection of boronsome (500 mg/kg, i.v.). Data are means ± SEM (n = 4 mice). Source data are provided as a Source Data file.

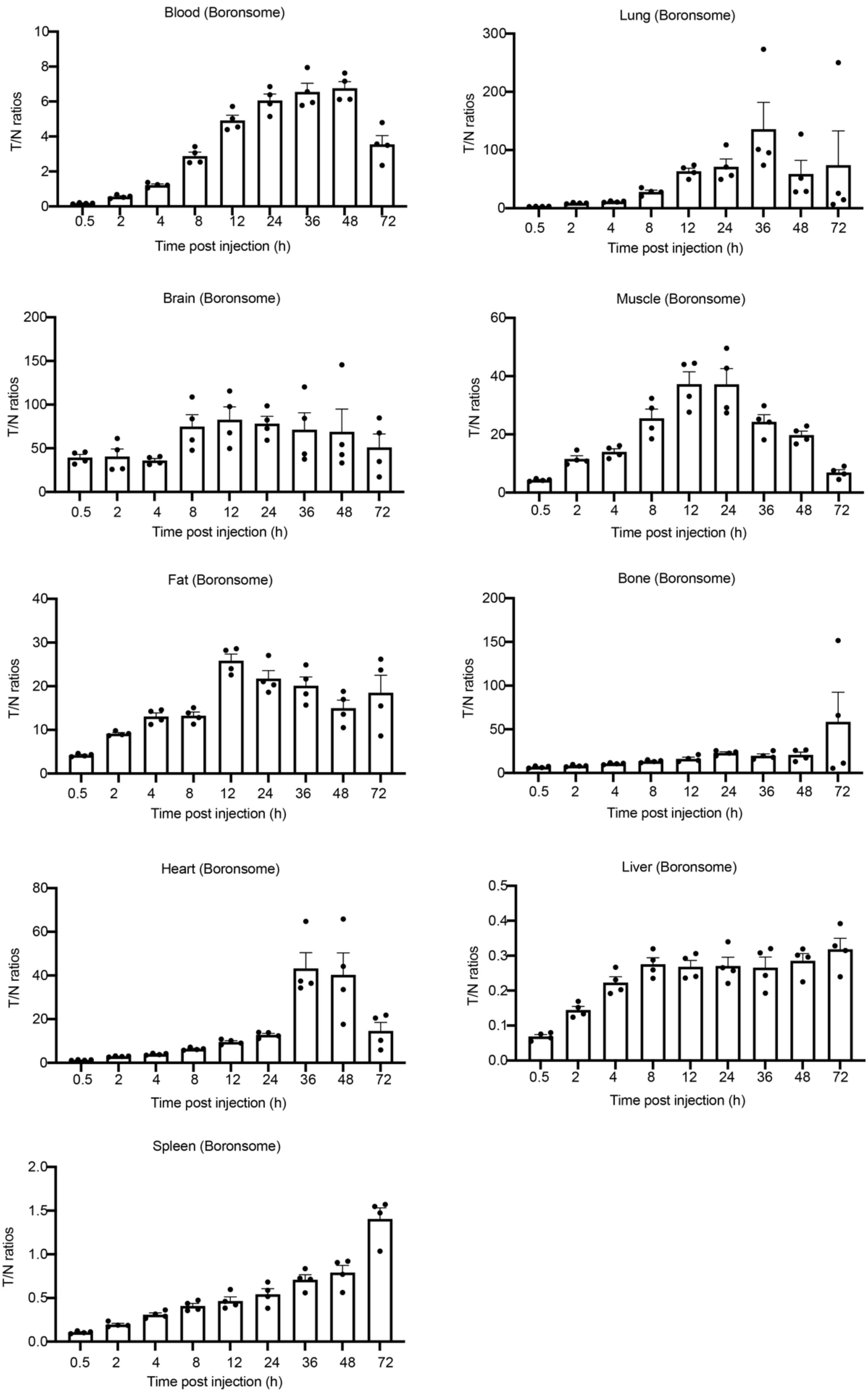

Supplementary Figure 7. Tumor-to-normal tissue ratio (T/N ratio) of blood, lung, brain, muscle, fat, bone, heart, liver, and spleen post once injection of boronsome (500 mg/kg, i.v.). Data are means ± SEM (n = 4 mice). Source data are provided as a Source Data file.

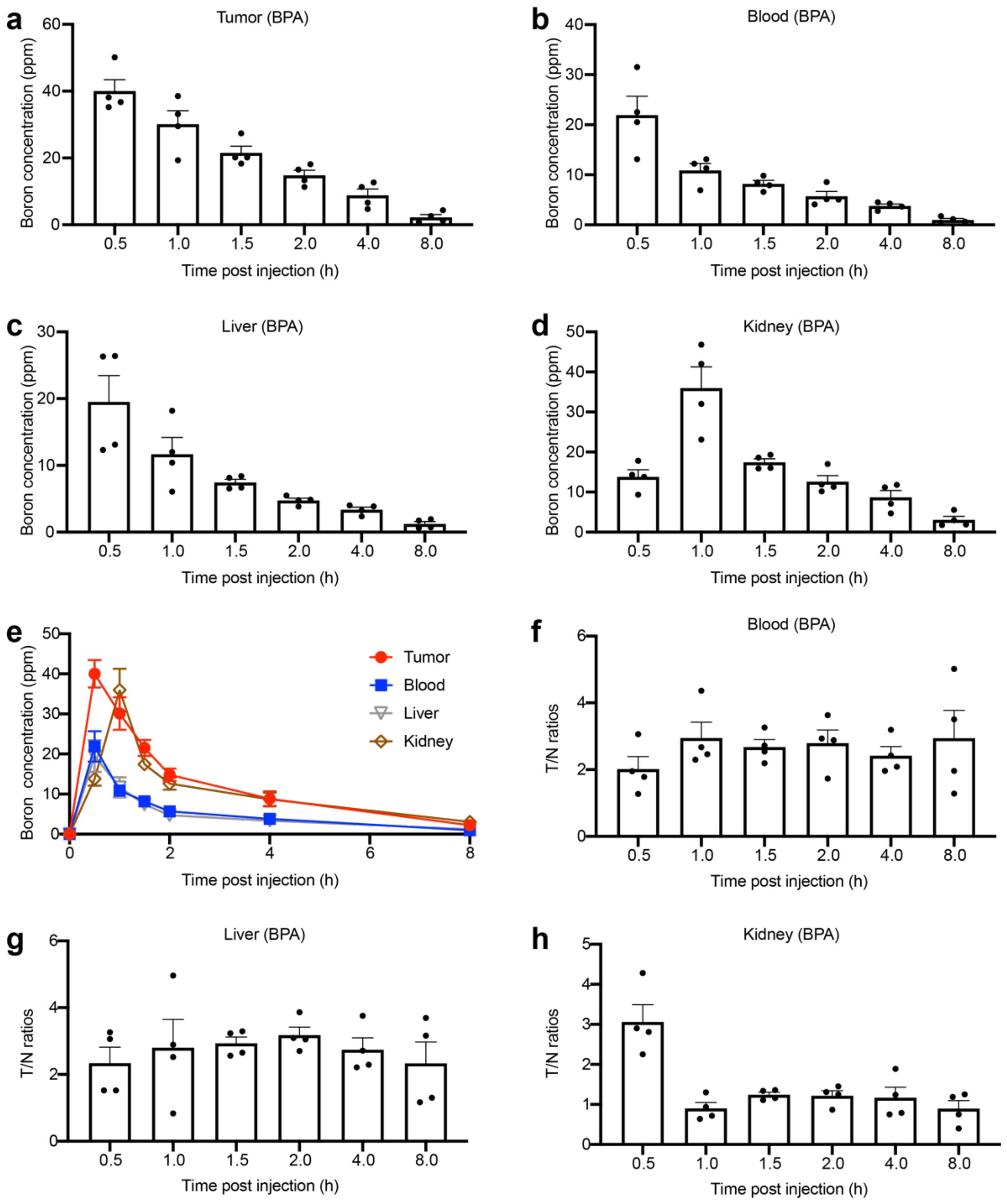

Supplementary Figure 8. Time-dependent boron concentration and T/N ratios of BPA in major organs. a-e Boron con in tumor, blood, liver, and kedney post once injection of BPA (500 mg/kg, i.v.). **f-h** Tumor-to-normal tissue ratio (T/N ratio) of blood, liver, and kidney post once injection of BPA (500 mg/kg, i.v.) Data are means ± SEM (n = 4 mice). Source data are provided as a Source Data file.

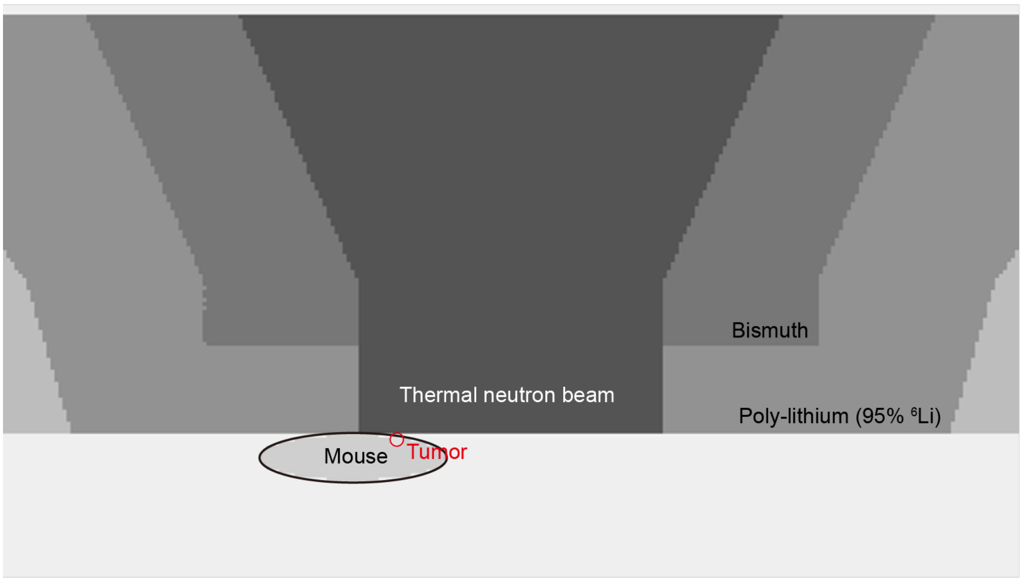

Supplementary Figure 9. SERA modeling.

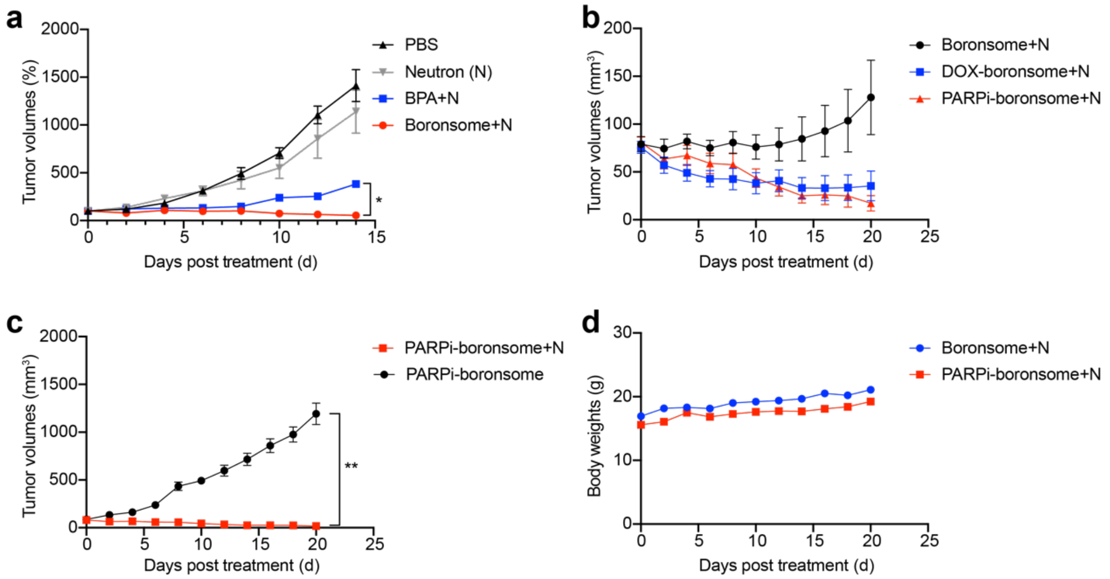

Supplementary Figure 10. Changes of tumor volumes and weights over time in different groups of mice. a Average tumor increase fold (n = 4) of each group of mice treated with PBS, neutron (N), BPA+N, and boronsome+N. Paired t-test, **p* = 0.0366. **b** Average tumor volumes (n = 9) of each group of mice treated with Boronsome+N, Dox-boronsome+N, and PARPi-boronsome+N. **c** Average tumor volumes (n = 9) of each group of mice treated with PARPi-boronsome and PARPi-boronsome+N. Paired t-test, ***p* = 0.0019. **d** Average body weights (n = 9) of each group of mice treated with boronsome+N or RARPi-boronsome+N. Data are means ± SEM. Source data are provided as a Source Data file.

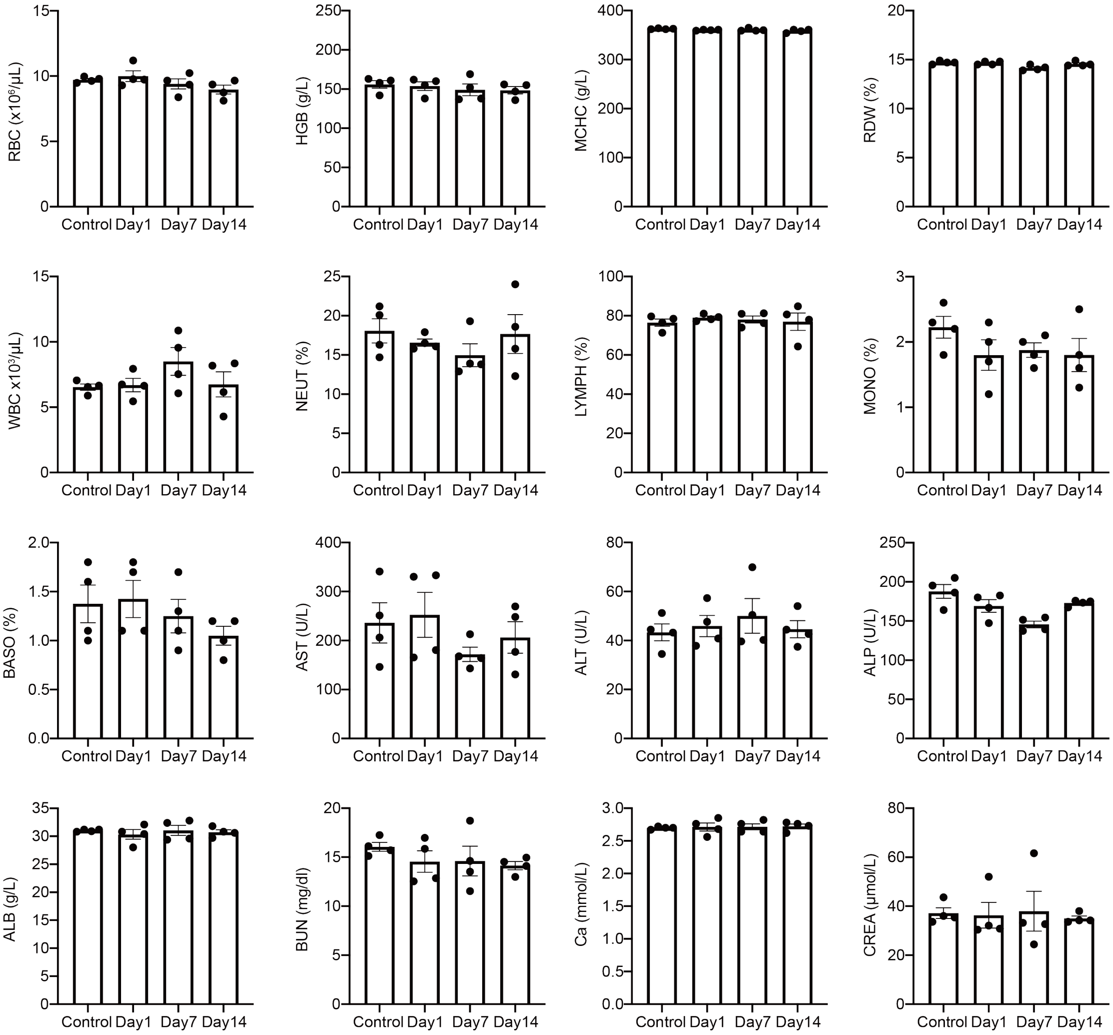

Supplementary Figure 11. The blood routine and biochemical test of 4T1 tumor-bearing mice intravenously injected with 500 mg/kg boronsome at day 1, day 7 and day 14 (n = 4). Data are means ± SEM. Source data are provided as a Source Data file.

NMR information

Compound **12**

1H NMR (400 MHz, chloroform-d): δ 4.16 (m, 2H), 3.75 (s, 1H), 2.97 (t, 2H), 2.39 (t, 2H), 1.91 (p, 2H), 1.27 (t, 3H), 0.80-3.40 (br, 10H) (85 % yield).

Compound **13**

1H NMR (400 MHz, chloroform-d): δ 4.13 (q, 2H), 3.73 (s, 1H), 2.90 (t, 2H), 2.29 (t, 2H), 1.49 (m, 10H), 1.26 (t, 3H), 0.80-3.40 (br, 10H) (86 % yield).

Compound **14**

1H NMR (400 MHz, chloroform-d): δ 4.12 (q, 2H), 3.72 (s, 1H), 2.90 (t, 2H), 2.28 (t, 2H), 1.57 (tt,4H), 1.26 (m, 17H), 0.80-3.40 (br, 10H) (89 % yield).

Compound **15**

1H NMR (400 MHz, chloroform-d): δ 4.12 (q, 2H), 3.72 (s, 1H), 2.90 (t, 2H), 2.28 (t, 2H), 1.58 (m,4H), 1.26 (m, 25H), 0.80-3.40 (br, 10H) (82 % yield).

Compound **16**

1H NMR (400 MHz, chloroform-d): δ 3.74 (s, 1H), 2.99 (t, 2H), 2.48 (t, 2H), 1.93 (p,2H), 0.80-3.40 (br, 10H) (78 % yield).

Compound **17**

1H NMR (400 MHz, chloroform-d): δ 3.72 (s, 1H), 2.90 (t, 2H), 2.36 (t, 2H), 1.60 (dp,4H), 1.34 (m, 6H), 0.80-3.40 (br, 10H) (79 % yield).

Compound **18**

1H NMR (400 MHz, chloroform-d): δ 3.72 (q, 1H), 2.90 (t,2H), 2.35 (t,2H), 1.59 (dp, 4H), 1.28 (m, 14H), 0.80-3.40 (br, 10H) (83 % yield).

Compound **19**

1H NMR (400 MHz, chloroform-d): δ 3.72 (s, 1H), 2.90 (t, 2H), 2.35 (t, 2H), 1.59 (dp,4H), 1.25 (s, 22H), 0.80-3.40 (br, 10H) (75 % yield).

Compound **21**

1H NMR (400 MHz, chloroform-d): δ 0.80-3.40 (br, 10H), 0.88 (m, 3H), 1.26 (s, 24H), 1.58 (d, 2H), 1.91 (p,2H), 2.31 (p, 2H), 2.44 (t, 2H), 2.98 (t, 2H), 3.33 (s, 9H), 3.77 (s, 1H), 4.02 (m, 7H), 4.44 (s, 2H) (38 % yield). $C_{30}H_{66}B_{10}NO_8PS$, m/z found 740.52.

Compound **22**

1H NMR (400 MHz, chloroform-d): δ 0.80-3.40 (br, 10H), 0.88 (t, 3H), 1.29 (d, 30H), 1.58 (dq, 6H), 2.33 (t, 4H), 2.90 (t, 2H), 3.34 (t, 9H), 3.73 (s, 1H), 3.79 (m, 5H), 4.09 (d, 2H), 4.41 (s, 2H) (33 % yield). $C_{34}H_{74}B_{10}NO_8PS$, m/z found 796.72.

Compound **23**

1H NMR (400 MHz, chloroform-d): δ 0.80-3.40 (br, 10H), 0.87 (t, 3H), 1.29 (d, 38H), 1.58 (m, 6H), 2.33 (t, 4H), 2.90 (t, 2H), 3.34 (t, 9H), 3.73 (s, 1H), 3.85 (m, 5H), 4.08 (dd, 2H), 4.41(s, 2H) (36 % yield). $C_{38}H_{82}B_{10}NO_8PS$, m/z found 852.69.

Compound **24**

1H NMR (400 MHz, chloroform-d): δ 0.80-3.40 (br, 10H), 0.87 (m, 3H), 1.25 (d, 46H), 1.58 (m, 6H), 2.33 (t, 4H), 2.90 (t, 2H), 3.31 (t, 9H), 3.72 (s, 1H), 3.88 (d, 5H), 4.08 (d, 2H), 4.36 (s, 2H) (22 % yield). $C_{42}H_{90}B_{10}NO_8PS$, m/z found 908.43.

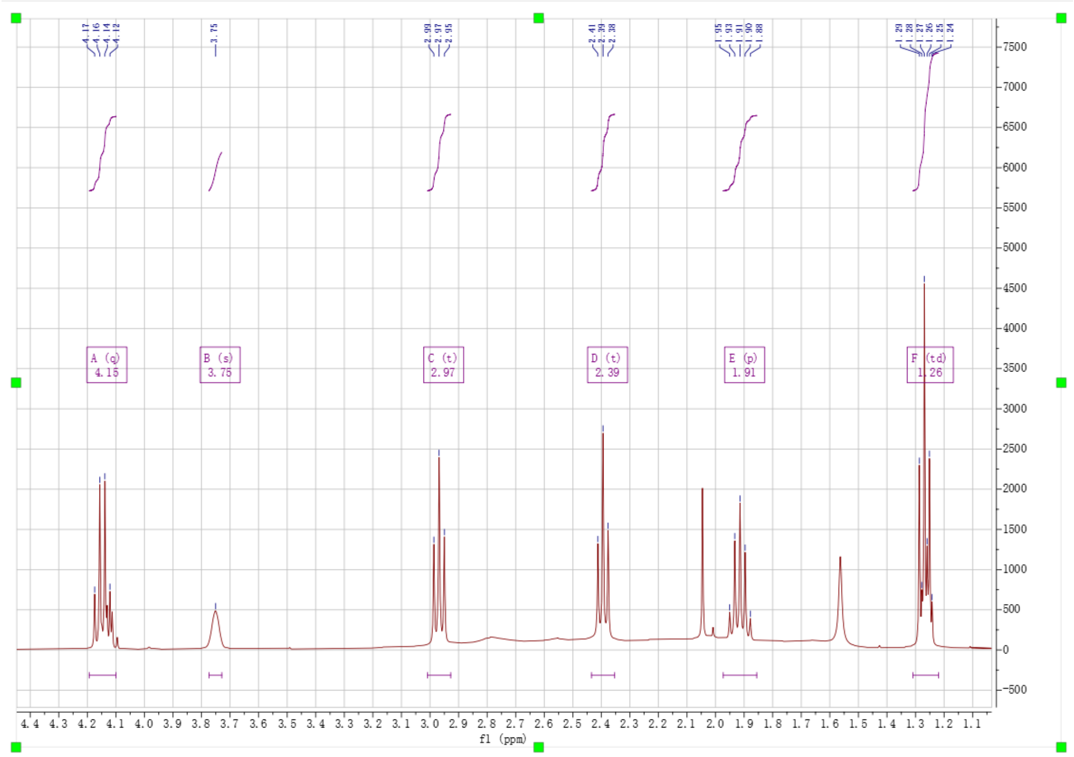

Supplementary Figure 12. ^{1}H-NMR spectrum of compound **12**.

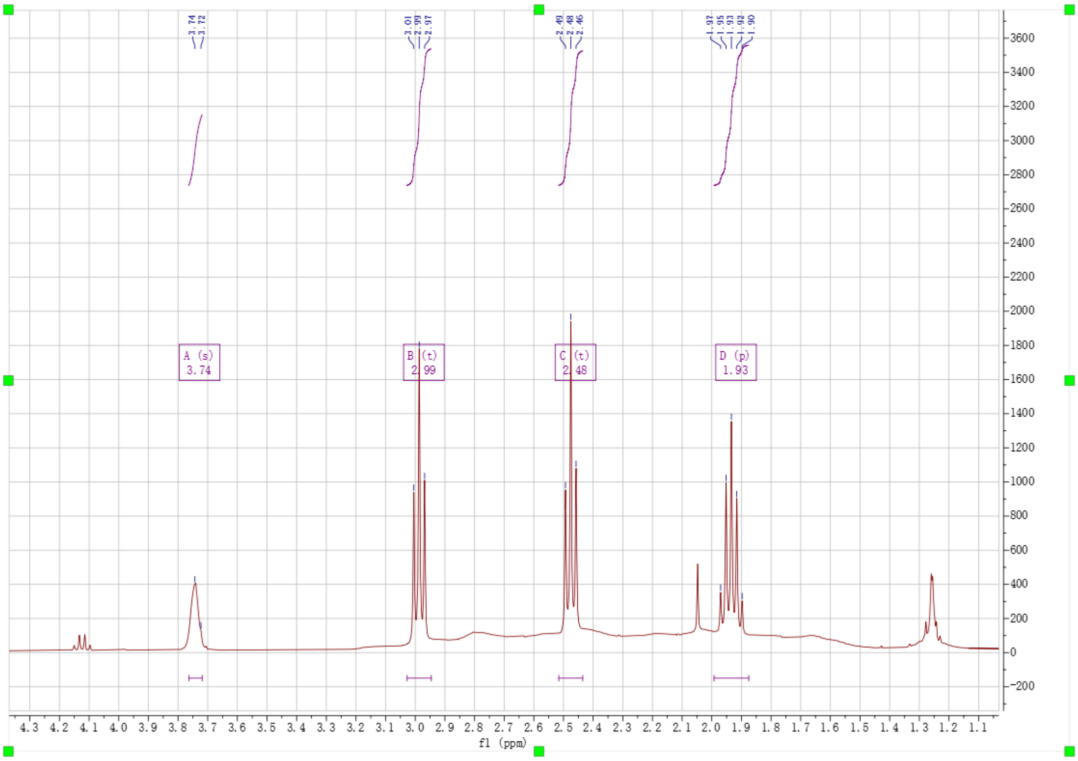

Supplementary Figure 13. ^{1}H-NMR spectrum of compound **13**.

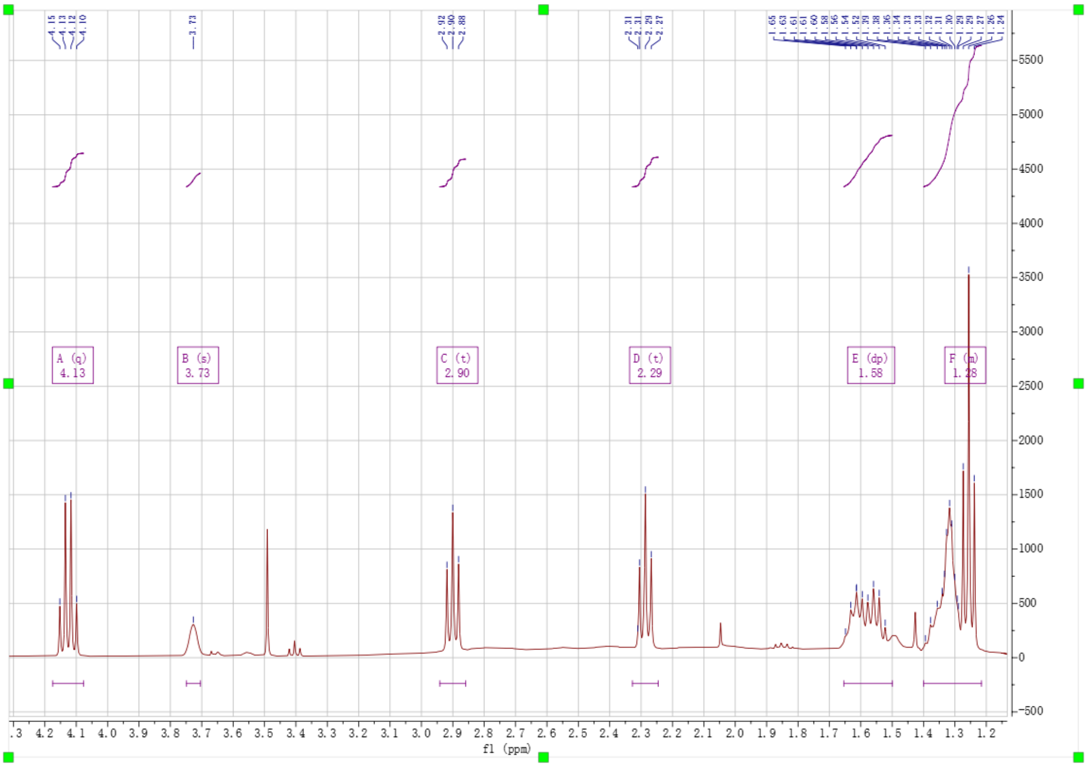

Supplementary Figure 14. ^{1}H-NMR spectrum of compound **14**.

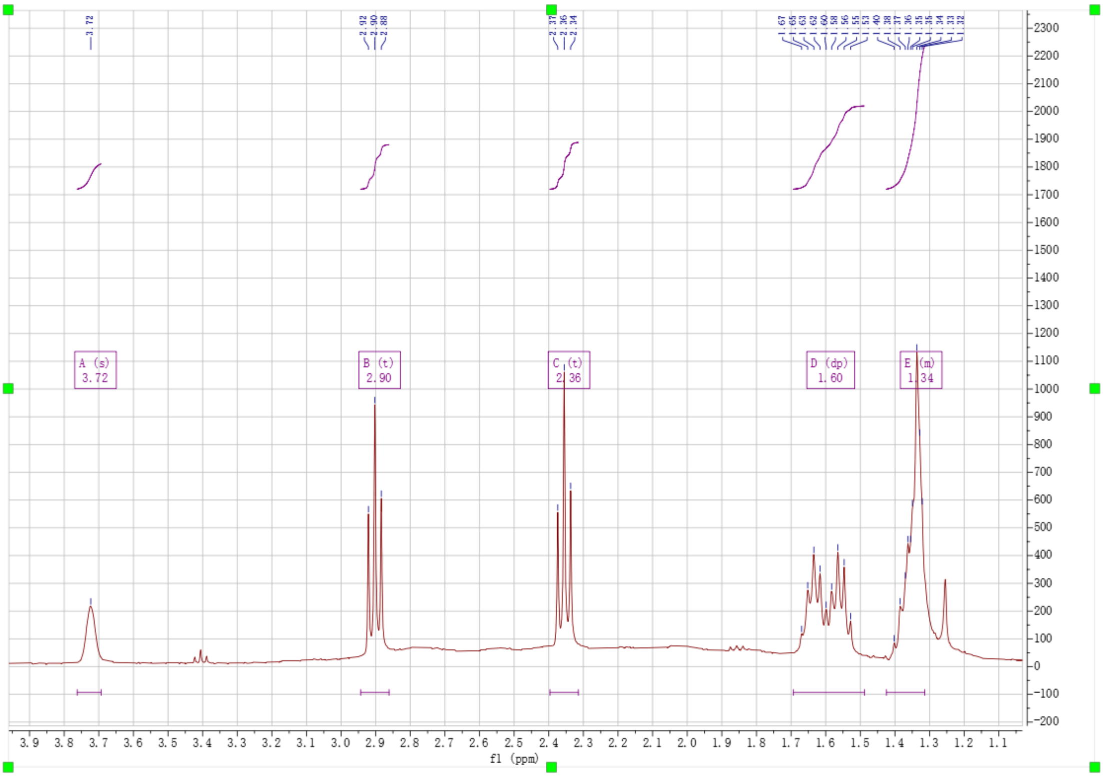

Supplementary Figure 15. ^{1}H-NMR spectrum of compound **15**.

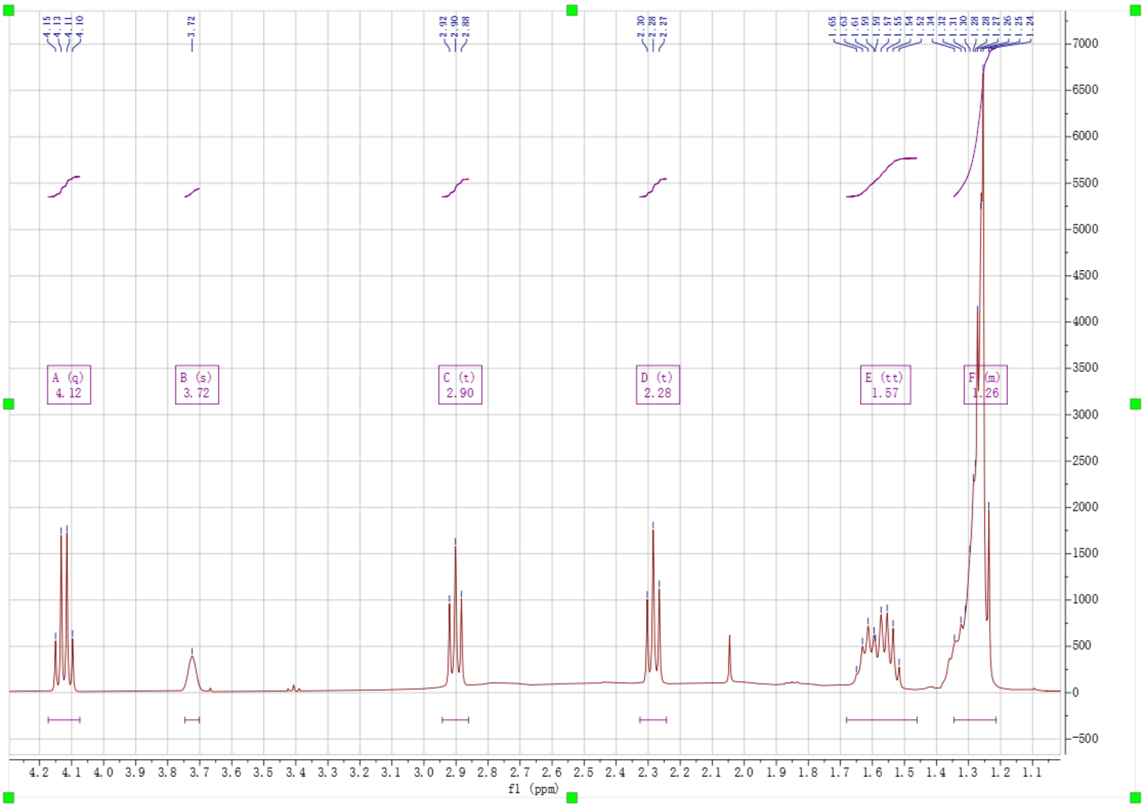

Supplementary Figure 16. ^{1}H-NMR spectrum of compound **16**.

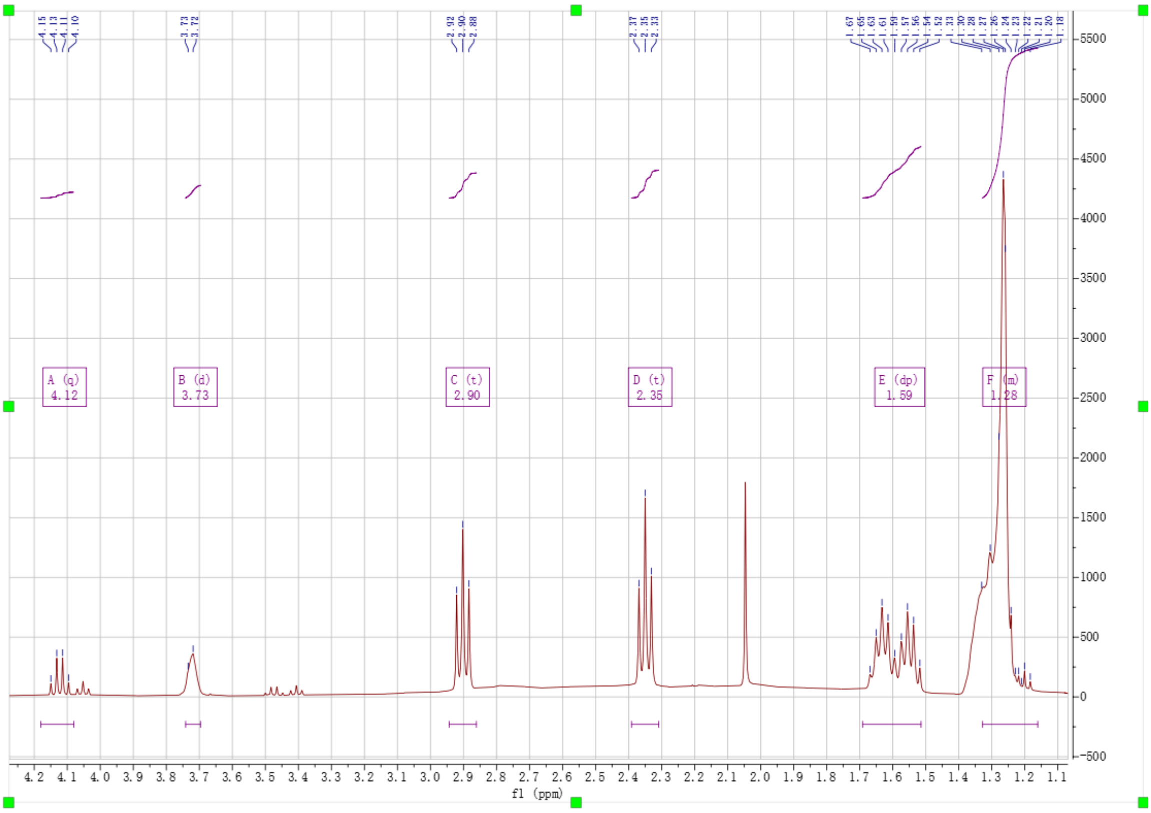

Supplementary Figure 17. ^{1}H-NMR spectrum of compound **17**.

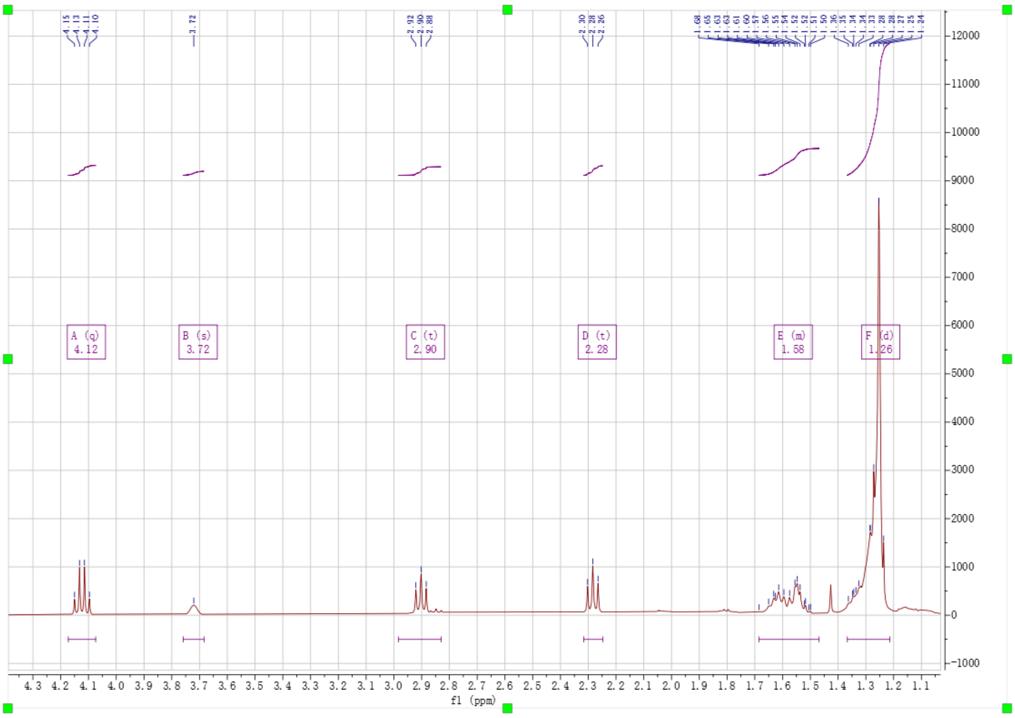

Supplementary Figure 18. ^{1}H-NMR spectrum of compound **18**.

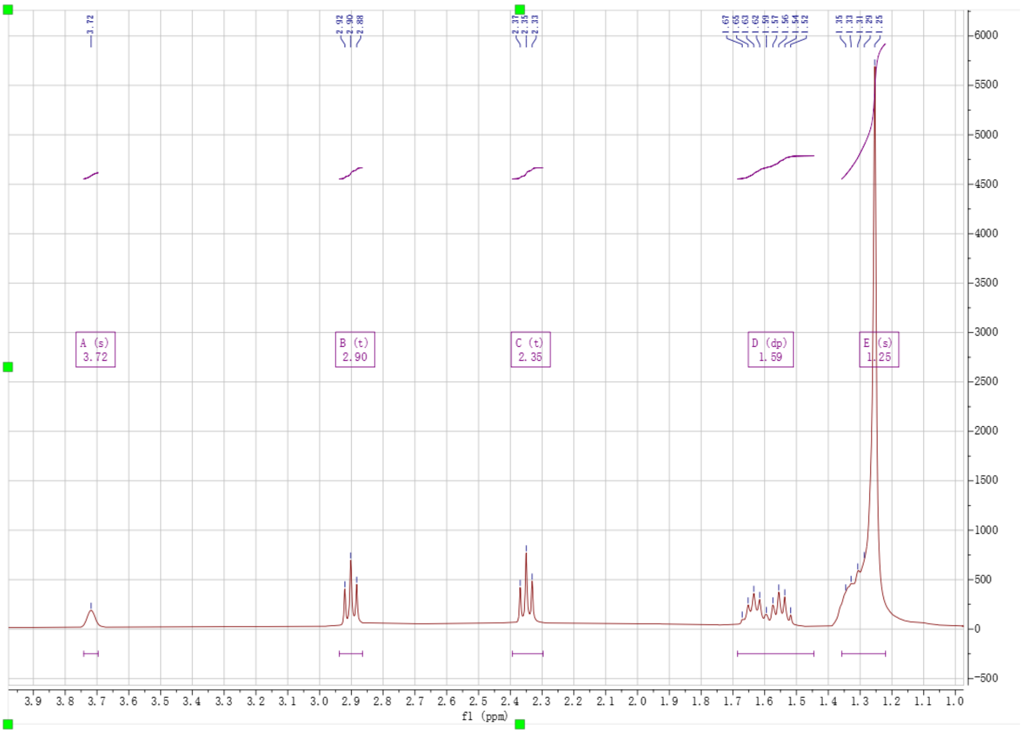

Supplementary Figure 19. ^{1}H-NMR spectrum of compound **19**.

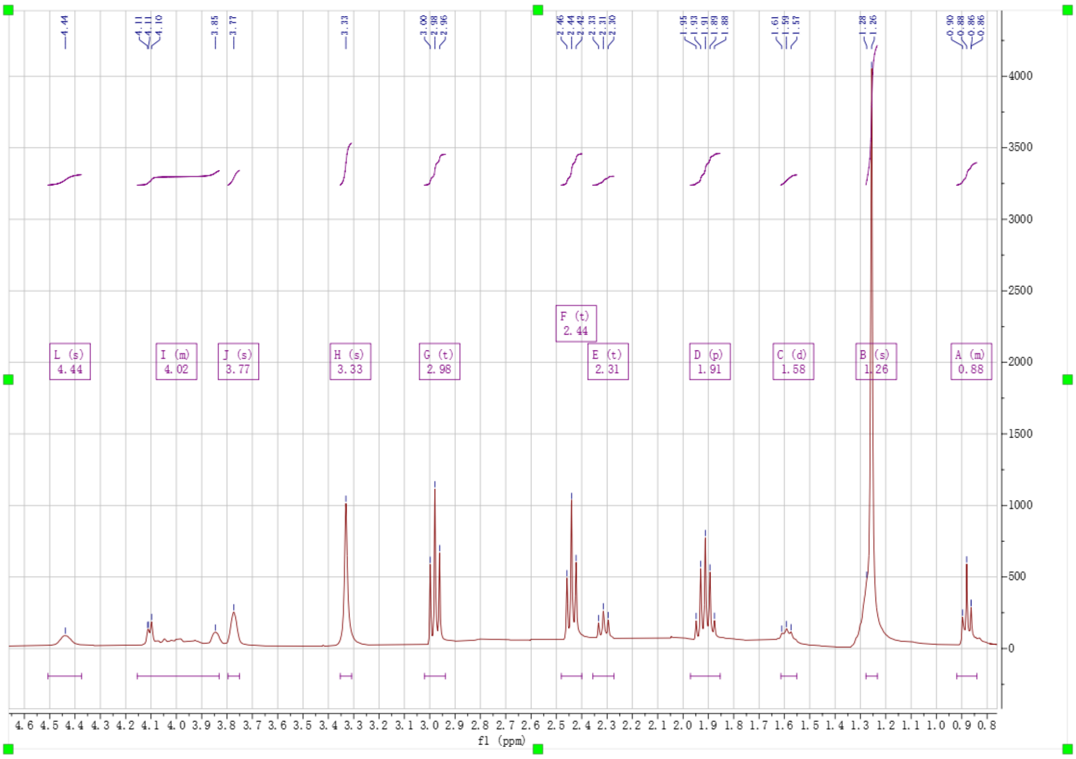

Supplementary Figure 20. ^{1}H-NMR spectrum of compound **21**.

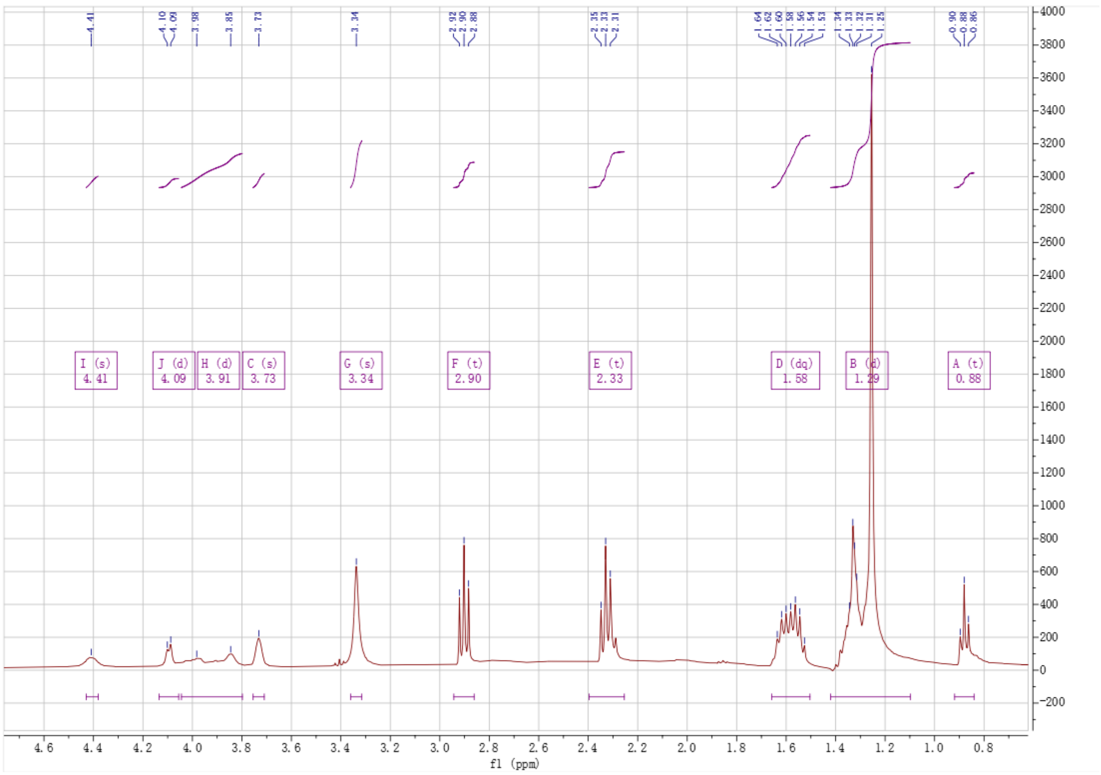

Supplementary Figure 21. ^{1}H-NMR spectrum of compound **22**.

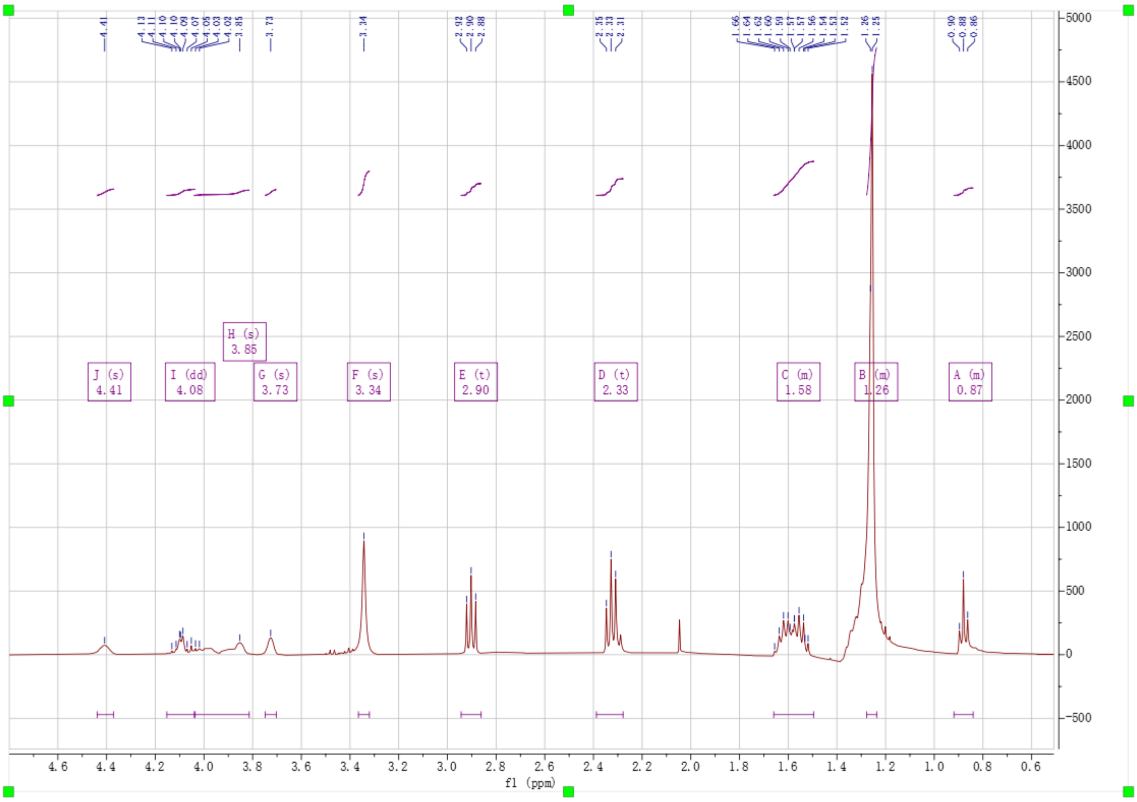

Supplementary Figure 22. ^{1}H-NMR spectrum of compound **23**.

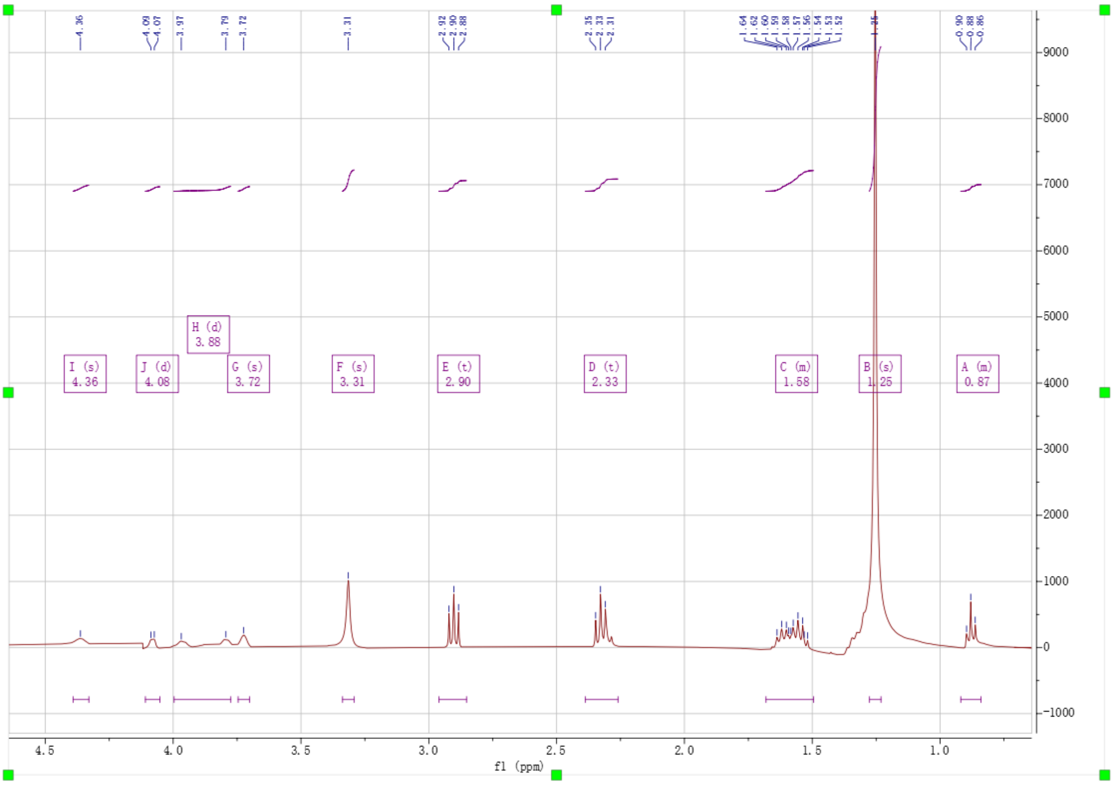

Supplementary Figure 23. ^{1}H-NMR spectrum of compound **24**.

HRMS information

Peking University Mass Spectrometry Sample Analysis Report

Analysis Info

Analysis Name	FTMS-22030070_Pos_20220307_000005.d	Acquisition Date	3/7/2022 10:32:16 AM
Sample	4C-COOH	Instrument	Bruker Solarix XR FTMS
Comment		Operator	Peking University

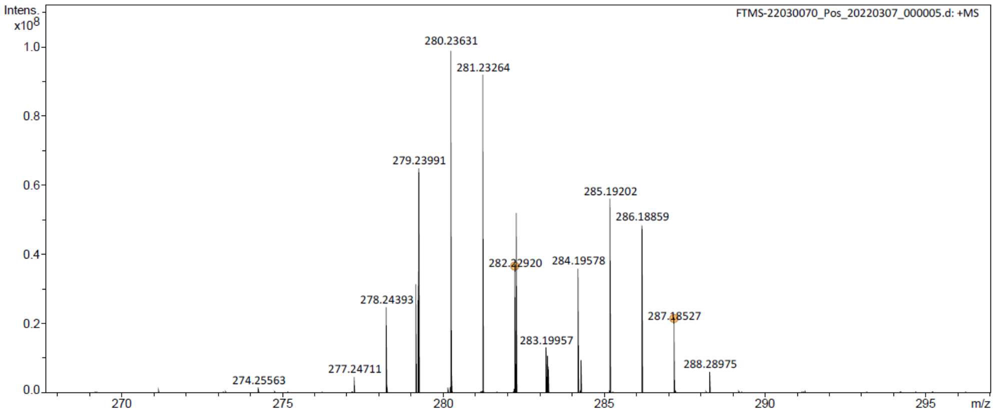

Meas. m/z	#	Ion Formula	Score	m/z	err [ppm]	Mean err [ppm]	mSigma	rdb	e⁻ Conf	N-Rule
282.229197	1	C6H22B10NO2S	100.00	282.229630	1.6	1.0	26.8	4.0	even	ok
287.185267	1	C6H18B10NaO2S	100.00	287.185025	-0.8	-1.0	32.1	5.0	even	ok

Supplementary Figure 24. High-resolution mass spectrum of compound **16**.

Peking University Mass Spectrometry Sample Analysis Report

Analysis Info

Analysis Name	FTMS-22030071_Pos_20220307_000006.d	Acquisition Date	3/7/2022 10:23:38 AM
Sample	8C-COOH	Instrument	Bruker Solarix XR FTMS
Comment		Operator	Peking University

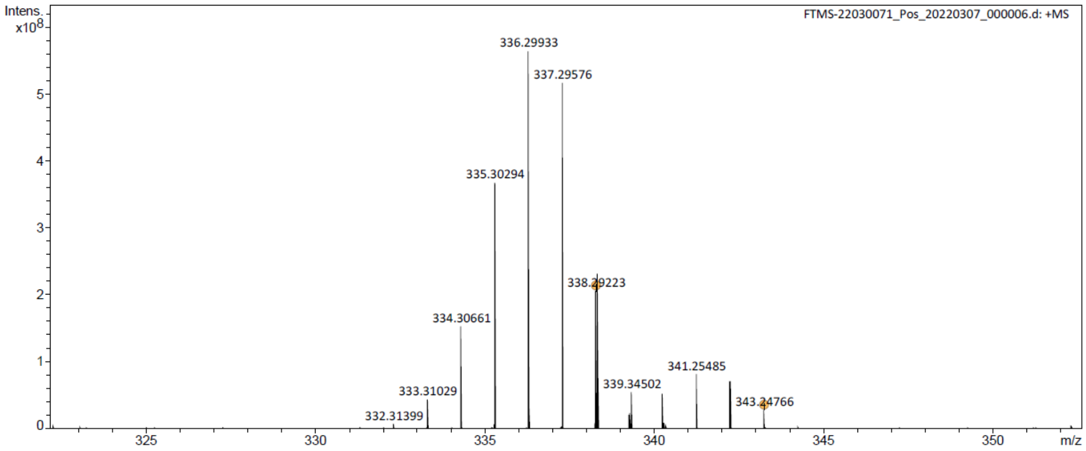

Meas. m/z	#	Ion Formula	Score	m/z	err [ppm]	Mean err [ppm]	mSigma	rdb	e⁻ Conf	N-Rule
338.292230	1	C10H30B10NO2S	100.00	338.292230	0.0	-0.5	33.6	4.0	even	ok
343.247662	1	C10H26B10NaO2S	100.00	343.247626	-0.1	-1.2	32.3	5.0	even	ok

Supplementary Figure 25. High-resolution mass spectrum of compound **17**.

Peking University Mass Spectrometry Sample Analysis Report

Analysis Info

Analysis Name FTMS-22030072_Pos_20220307_000001.d
Sample 12CCOOH
Comment

Acquisition Date 3/7/2022 10:12:34 AM
Instrument Bruker Solarix XR FTMS
Operator Peking University

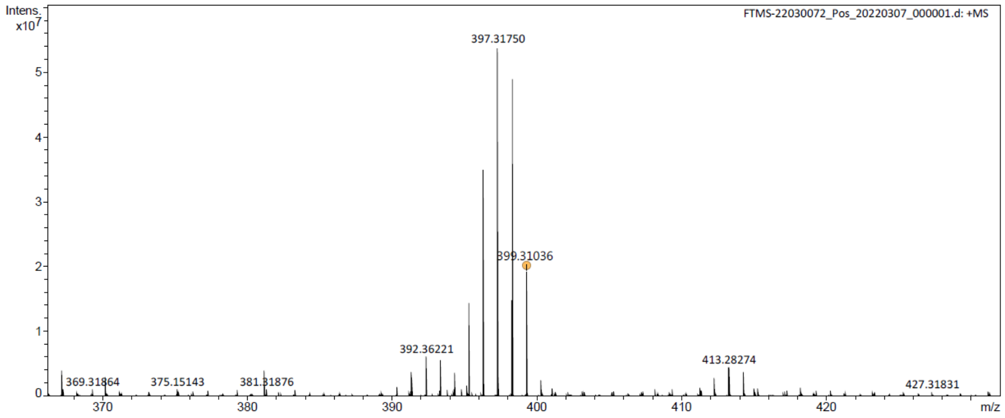

Meas. m/z	#	Ion Formula	Score	m/z	err [ppm]	Mean err [ppm]	mSigma	rdb	e⁻ Conf	N-Rule
399.310365	1	C14H34B10NaO2S	100.00	399.310226	-0.3	-2.2	44.0	5.0	even	ok

Supplementary Figure 26. High-resolution mass spectrum of compound **18**.

Peking University Mass Spectrometry Sample Analysis Report

Analysis Info

Analysis Name FTMS-22030073_Pos_20220307_000005.d
Sample 16C-COOH
Comment

Acquisition Date 3/7/2022 10:07:10 AM
Instrument Bruker Solarix XR FTMS
Operator Peking University

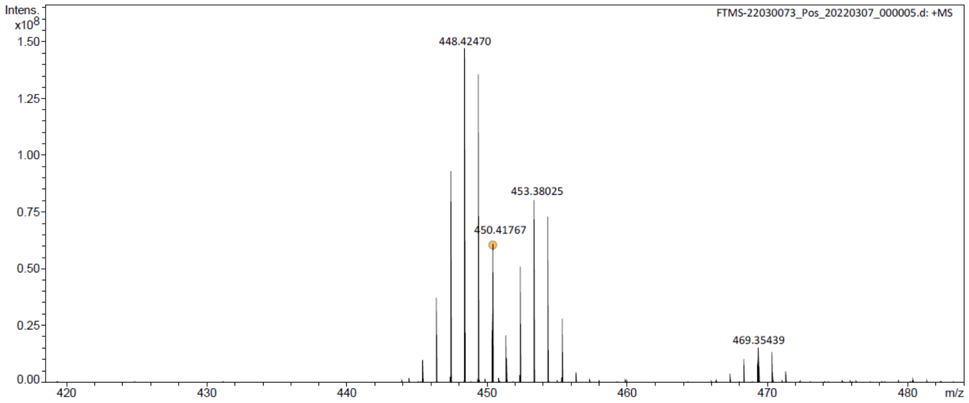

Meas. m/z	#	Ion Formula	Score	m/z	err [ppm]	Mean err [ppm]	mSigma	rdb	e⁻ Conf	N-Rule
450.417669	1	C18H46B10NO2S	100.00	450.417431	0.3	-0.1	18.5	4.0	even	ok

Supplementary Figure 27. High-resolution mass spectrum of compound **19**.
