# Supplementary material for: Boron encapsulated in a liposome can be used for combinational neutron capture therapy
Source: Nat Commun. 2022 Apr 19;13:2143. doi: 10.1038/s41467-022-29780-w (PMC9018847; doi:10.1038/s41467-022-29780-w)
Supplement: Supplementary file 3 — Reporting Summary [file 41467_2022_29780_MOESM3_ESM.pdf]

nature portfolio

Corresponding author(s): Zhibo Liu

Last updated by author(s): Mar 16, 2022

Reporting Summary

Nature Portfolio wishes to improve the reproducibility of the work that we publish. This form provides structure for consistency and transparency in reporting. For further information on Nature Portfolio policies, see our Editorial Policies and the Editorial Policy Checklist.

Statistics

For all statistical analyses, confirm that the following items are present in the figure legend, table legend, main text, or Methods section.

n/a	Confirmed	
☐	☒	The exact sample size (*n*) for each experimental group/condition, given as a discrete number and unit of measurement
☐	☒	A statement on whether measurements were taken from distinct samples or whether the same sample was measured repeatedly
☐	☒	The statistical test(s) used AND whether they are one- or two-sided<br>*Only common tests should be described solely by name; describe more complex techniques in the Methods section.*
☒	☐	A description of all covariates tested
☒	☐	A description of any assumptions or corrections, such as tests of normality and adjustment for multiple comparisons
☐	☒	A full description of the statistical parameters including central tendency (e.g. means) or other basic estimates (e.g. regression coefficient) AND variation (e.g. standard deviation) or associated estimates of uncertainty (e.g. confidence intervals)
☐	☒	For null hypothesis testing, the test statistic (e.g. *F*, *t*, *r*) with confidence intervals, effect sizes, degrees of freedom and *P* value noted<br>*Give P values as exact values whenever suitable.*
☒	☐	For Bayesian analysis, information on the choice of priors and Markov chain Monte Carlo settings
☐	☒	For hierarchical and complex designs, identification of the appropriate level for tests and full reporting of outcomes
☒	☐	Estimates of effect sizes (e.g. Cohen's *d*, Pearson's *r*), indicating how they were calculated

Our web collection on statistics for biologists contains articles on many of the points above.

Software and code

Policy information about availability of computer code

Data collection	Characterization of boronsome: Zetasizer Nano ZS system (Malvern Instruments Ltd), TEM operated at 200 kV (JEM-2100, JEOL); Radio-TLC: Scan-RAM (LabLogic); 3D structures modeling and MD simulation: Spartan 14, Gromacs 2018, ATB 3.0; The Monte Carlo computation-based dose calculation: SERA 1C1; ICP-OES: microwave accelerated reaction system (Mars, CEM), Optima 7000 DV (PerkinElmer); Microscope: laser scanning confocal microscope (Nikon); PET/CT imaging: Nanoscan PET-CT 122s (Mediso Medical Imaging Systems); H&E staining: 3Dhistech (3DHISTECH)
Data analysis	3D structures modeling and MD simulation: VMD v1.9.3; Immunofluorescence staining: ImageJ v1.53a; Statistical analysis: GraphPad Prism v8; PET/CT imaging: InterViewTM FUSION v3.09 (Mediso Medical Imaging Systems); H&E staining: CaseViewer v2.4

For manuscripts utilizing custom algorithms or software that are central to the research but not yet described in published literature, software must be made available to editors and reviewers. We strongly encourage code deposition in a community repository (e.g. GitHub). See the Nature Portfolio guidelines for submitting code & software for further information.

Data

Policy information about availability of data

All manuscripts must include a data availability statement. This statement should provide the following information, where applicable:

- Accession codes, unique identifiers, or web links for publicly available datasets
- A description of any restrictions on data availability
- For clinical datasets or third party data, please ensure that the statement adheres to our policy

All data generated or analysed during this study are included in this published article (and its supplementary information files).

Field-specific reporting

Please select the one below that is the best fit for your research. If you are not sure, read the appropriate sections before making your selection.

☒ Life sciences ☐ Behavioural & social sciences ☐ Ecological, evolutionary & environmental sciences

For a reference copy of the document with all sections, see nature.com/documents/nr-reporting-summary-flat.pdf

Life sciences study design

All studies must disclose on these points even when the disclosure is negative.

Sample size	Exact sample size is stated in the figure legend. In the animal experiments to detect tumour growth, for individual analyses n is 9 in each group. For all other experiment, for individual analyses n is 3, 4, or 6, selected on the basis that the variability between estimates is sufficiently small to provide significant differences between test samples in these studies.
Data exclusions	No data were excluded from the analyses
Replication	All results were repeated at least three times independently with similar results.
Randomization	Randomization was applied following these two criteria: the probability of assignment to any of the experimental groups is equal for each subject and the assignment of one subject to a group does not affect the assignment of any other subject to that same group
Blinding	Proper blinding was applied during the data collection and analysis

Reporting for specific materials, systems and methods

We require information from authors about some types of materials, experimental systems and methods used in many studies. Here, indicate whether each material, system or method listed is relevant to your study. If you are not sure if a list item applies to your research, read the appropriate section before selecting a response.

Materials & experimental systems

n/a	Involved in the study	
☐	☒	Antibodies
☐	☒	Eukaryotic cell lines
☒	☐	Palaeontology and archaeology
☐	☒	Animals and other organisms
☒	☐	Human research participants
☒	☐	Clinical data
☒	☐	Dual use research of concern

Methods

n/a	Involved in the study	
☒	☐	ChIP-seq
☒	☐	Flow cytometry
☒	☐	MRI-based neuroimaging

Antibodies

Antibodies used	anti-γH2AX antibody (1:200, abcam, ab81299, EP854(2)Y,) ; goat anti-rabbit IgG H&L (1:1000, Alexa Fluor® 488, abcam, ab150077, Polyclonal)
Validation	anti-γH2AX antibody: Rabbit anti-mouse antibody, Immunofluorescence. IgG H&L: goat anti-rabbit, Immunofluorescence.

Eukaryotic cell lines

Policy information about cell lines

Cell line source(s)	The 4T1 cell lines (3101MOUSCSP5056) were obtained from National Infrastructure of Cell Line Resource (Beijing, China).
Authentication	Identity of the cell lines were frequently checked by their morphological features but have not been authenticated by short tandem repeat (STR) profiling.
Mycoplasma contamination	All cell lines tested negative for mycoplasma contamination.
Commonly misidentified lines (See ICLAC register)	No commonly misidentified cell line was used in the study

Animals and other organisms

Policy information about studies involving animals; ARRIVE guidelines recommended for reporting animal research

Laboratory animals	6-8-weeks female BALB/c mice were obtained from Beijing Vital River Laboratory Animal Technology Co, Ltd. and maintained under specific pathogen-free facility (SPF) conditions with a 12 light/12 dark cycle, and free access to food and water. Mice were housed under temperature of 24 ± 2°C, and humidity of 50 ± 10%.
Wild animals	The study did not involve wild animals
Field-collected samples	The study did not involve samples collected from the field
Ethics oversight	All animal care and experimental procedure were performed by following the animal protocols (CCME-LiuZB-2) approved by the ethics committee of Peking University

Note that full information on the approval of the study protocol must also be provided in the manuscript.
